# The power of neutralization: the critical step for the accurate antimicrobial potential of plasma-activated water

**DOI:** 10.3389/fmicb.2026.1774713

**Published:** 2026-02-20

**Authors:** Manca Lunder, Sebastian Dahle, Borut Poljšak, Rok Fink

**Affiliations:** 1 Faculty of Health Sciences, University of Ljubljana, Ljubljana, Slovenia; 2 Faculty of Biotechnical, University of Ljubljana, Ljubljana, Slovenia; 3 Laboratory of Oxidative Stress Research, Faculty of Health Sciences, University of Ljubljana, Ljubljana, Slovenia

**Keywords:** antibacterial, cold atmospheric plasma, disinfection, neutralization, plasma activated water

## Abstract

Cold atmospheric plasma (CAP) has emerged as a promising alternative technology for water disinfection due to its strong antimicrobial activity mediated by plasma-activated water (PAW). In this study, CAP generated using a flow-through dielectric barrier discharge (DBD) reactor was evaluated for its antimicrobial efficacy against *Escherichia coli* and *Staphylococcus aureus* in model hard water, with particular emphasis on post-treatment reactivity and the need for neutralization in antimicrobial testing. CAP treatment for 3 min resulted in log reductions of 1.09 for *E. coli* and 3.27 for *S. aureus*, confirming effective microbial inactivation. Storage of PAW at 4°C for 24 h led to complete inactivation of both strains, demonstrating persistent antimicrobial activity driven by long-lived reactive oxygen and nitrogen species (RONS). Quantification of hydrogen peroxide, ozone, nitrite, and nitrate revealed significant depletion of ozone and hydrogen peroxide during storage, particularly in the presence of bacteria, indicating ongoing chemical–biological interactions. Nitrite and nitrate remained comparatively stable, suggesting a secondary or synergistic role in prolonged antimicrobial effects. The persistence of PAW activity highlighted the necessity of immediate neutralization to avoid overestimation of antimicrobial efficacy. Several chemical neutralizers recommended in standardized antimicrobial testing protocols were evaluated, with a combined “Mix” formulation (PBS, NaCl with tryptone, polysorbate 80, lecithin, and sodium thiosulphate) providing the most effective quenching of residual RONS while remaining non-toxic to bacteria. In parallel, the influence of solid culture media on bacterial recovery was assessed. We recommend combining the Mix neutralizer with non-selective or mildly selective media (NEA for *E. coli* and NMSA for *S. aureus*) to improve reproducibility and reliability in PAW antimicrobial testing. These findings contribute to methodological standardization and support the development of CAP-based water disinfection technologies.

## Graphical Abstract

**Figure:**
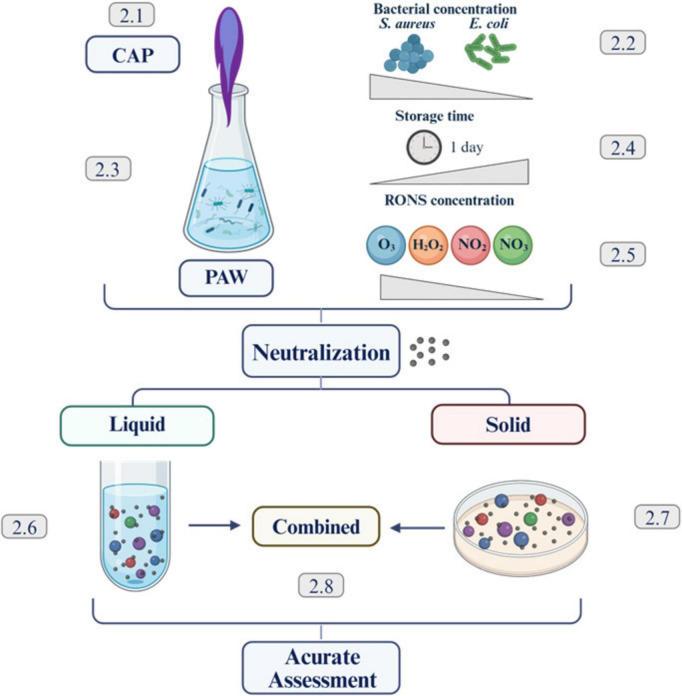
Evaluation of different neutralizers for assessing antimicrobial potential of PAW.

## 1 Introduction

Accurate and reliable results are fundamental to producing valid microbiological findings (Karadayi et al., 2017). A critical step in properly evaluating the efficacy of biocidal products is the neutralization of antimicrobial activity (Eissa, 2016), as the presence of inhibitory substances in culture media can suppress the growth of viable microorganisms, leading to misleading or inaccurate laboratory results and underestimated risk (Mehrgan et al., 2006). The reliability of microbiological analyses of liquid samples largely depends on ensuring that each sample truly reflects its condition at the time of collection. Therefore, during water sampling, any residual biocidal activity must be completely neutralized to prevent continued antimicrobial effects (Lai et al., 2021). The antimicrobial activity of agents can be neutralized through several approaches, including filtration, and chemical neutralization (Katerji et al., 2023). Filtration aims to remove antimicrobials from suspension but may be limited by adsorption of the agents to either the membrane filter or microbial cells, thereby reducing recovery. Lastly, residual antimicrobials can be inhibited by chemical neutralization, using a wide variety of neutralizers (Mehrgan et al., 2006). The chemical eq. liquid neutralization method is also the preferred approach for conducting antimicrobial effectiveness tests by the United States Pharmacopeia (USP) (Katerji et al., 2023). A validated protocol involves applying an antimicrobial agent on bacterial suspension and mixing it with a neutralizing agent with for an appropriate contact period (typically 5–10 min), before applying the mixture to the growth medium (Eissa, 2016). Standardized methods such as EN 1276, ASTM E1054, and USP guidelines recommend specific neutralizers tailored to different antimicrobial compounds. For common agents such as quaternary ammonium compounds, chlorine, hydrogen peroxide, aldehydes, and alcohols, these standards clearly specify neutralizer formulations containing lecithin, saponin, polysorbate 80, sodium thiosulfate, glycine, and magnesium ions (ASTM, 2002; The United States Pharmacopeial Convention, 2012; EN 1276, 2020). Additionally, EN 1276 (2020) notes that sodium chloride supplemented with tryptone may also serve as a neutralizing rinse solution in membrane filtration methods.

While neutralization strategies for conventional chemical antimicrobials are well established, emerging disinfection technologies pose new challenges that require tailored approaches. Among these, Cold Atmospheric Plasma (CAP) has gained attention as a promising alternative due to its strong antimicrobial potential and environmentally friendly nature (Zhao et al., 2020b; Lunder et al., 2025). Plasma is a partially or fully ionized gas composed of various reactive species, including reactive oxygen and nitrogen species (RONS), electrons, positive and negative ions, free radicals, gas atoms and molecules in different energy states, that are responsible for microbial inactivation (Liao et al., 2018). When CAP interacts with water, it triggers a cascade of chemical reactions, resulting in the formation of Plasma Activated Water (PAW) (Machala et al., 2019), consisting of numerous aqueous RONS (Zhou et al., 2020). Notably, these can be classified into short- and long-lasting species (Lunder et al., 2025). Short-lived species, such as hydroxyl radicals and hydrated electrons (Zhao et al., 2020a), exist only briefly—hydroxyl radicals, for instance, have a residence time of about 150 ps at the gas–water interface (Wick and Dang, 2007). In contrast, long-lived species such as ozone and hydrogen peroxide can persist for several to dozens of minutes and can diffuse into the liquid phase (Bruggeman et al., 2016; Wong et al., 2023), where they may remain stable for weeks or even months (Shen et al., 2016; Tsoukou et al., 2020). These long-lived RONS can also undergo secondary post-treatment reactions, generating additional, more stable species. A detailed discussion of RONS chemistry is beyond the scope of this manuscript, and readers are referred to our previous work for a comprehensive overview (Lunder et al., 2025). Consequently, when evaluating or standardizing antimicrobial assays involving PAW, it is essential to neutralize these long-lived oxidants rather than short-lived RONS, as the latter decay spontaneously before they can interfere with downstream analyses.

CAP treatment of contaminated water affects the microbial cells via cell wall permeabilization, penetration of reactive species and chemical actions inside the cell (Zimmermann et al., 2012). CAP exposure induces oxidative stress in microbial cells, damaging DNA, proteins, and lipids while compromising membrane integrity, which facilitates further RONS infiltration and ultimately cell death (Xu et al., 2020). Furthermore, the long-lasting antibacterial effects of PAW itself are primarily attributed to stable, long-lived reactive species (Traylor et al., 2011; Shen et al., 2016; Zhang et al., 2024), which have been shown to persist for at least 30 days while maintaining antimicrobial activity (Shen et al., 2016). Consequently, to prevent continued microbial inactivation after sampling, it is necessary to neutralize CAP-derived long-lived RONS before microbiological analysis. This requirement becomes clearer when compared with classical disinfection approaches. For example, chlorine-based disinfectants generate a pronounced residual effect, as free chlorine or chloramines remain active in the treated medium and continue to inactivate microorganisms long after the initial exposure. Therefore, neutralizers such as sodium thiosulfate must be added prior to microbiological enumeration to quench any remaining disinfectant and prevent overestimation of antimicrobial efficacy (Dotson et al., 2012; Wang et al., 2024). In contrast, UV-based disinfection does not introduce any chemical agents into the medium and produces no residual antimicrobial activity (Dotson et al., 2012). Once UV irradiation ceases, the inactivation process stops immediately, eliminating the need for subsequent neutralization steps. This distinction highlights why neutralization is essential for PAW and other treatments that generate long-lived reactive species, but unnecessary for methods that lack residual effects. It should be noted, however, that such neutralization primarily targets long-lived species and may not fully eliminate the effects of freshly produced PAW, in which short-lived reactive species can still contribute to antimicrobial activity.

In most reviewed studies where PAW was tested (Table 1), solely dilution without neutralization was used (Ikawa et al., 2010; Shen et al., 2016; Royintarat et al., 2019; Baek et al., 2020). In some studies, phosphate-buffered saline (PBS) (Cai et al., 2023; Boopathy et al., 2024), peptone saline diluent (Han et al., 2016), or peptone water (Hadinoto et al., 2021) are used as the dilution medium. Only a few studies have emphasized the neutralization step after PAW treatment and have employed and validated chemical neutralizers (Hummert et al., 2023), for example, used neutralization solution containing 3-L-α-phosphatidylcholine (0.3%), Tween 80 (3%), sodium thiosulphate (0.5%), L-histidine (0.1%), and saponin (3%) for neutralization of PAW, modifying the DIN EN 1040 standard for testing chemical disinfectants and antiseptics. On the other hand, Xu et al. (2020) removed RONS by centrifuging the sample and resuspending bacterial cells in PBS. Meanwhile, some of the studies skipped the neutralization step altogether and applied PAW directly onto the incubating solid medium (Machala et al., 2019; Schmidt et al., 2019; Maybin et al., 2023). Most studies use non-selective media such as Tryptic Soy Agar (TSA), Nutrient Agar (NA), or Luria Bertani Agar (LBA). However, in some cases, selective agars are used because PAW-producing devices cannot guarantee sterile conditions.

**TABLE 1:** The use of neutralization in PAW antimicrobial testing studies.

Study	Neutralizer	Growth media	Microorganism
Boopathy et al. (2024)	Dilution in PBS	LBA	*Salmonella enterica*
Nguyen et al. (2020)	/	MPN	*E. coli*
Wang and Salvi (2021)	/	TSA	*E. coli* and *Listeria innocua*
Huang et al. (2018)	Dilution in PBS	BHIA, SSA, BPA	*Salmonella typhimurium* and *S. aureus*
Cai et al. (2023)	Dilution in PBS	PCA	*Pseudomonas aeruginosa* and *Listeria monocytogenes*
Hadinoto et al. (2021)	Dilution in peptone water	NA	*E. coli* and *Salmonella typhimurium*
Abdo et al. (2023)	/	NA	*Salmonella typhimurium* field isolates
Baek et al. (2020)	/	TSA	*Listeria monocytogenes* and *E. coli*
Han et al. (2016)	Dilution in peptone saline diluent	TSA	*E. coli* and *S. aureus*
Hummert et al. (2023)	Neutralizer (3-L-α-phosphatidylcholine, Tween 80, sodium thiosulphate, L-histidine, and saponin)	LBA	*Pseudomonas aeruginosa*
Ikawa et al. (2010)	/	LBA	*E. coli*
Machala et al. (2019)	/	LBA	*E. coli*
Maybin et al. (2023)	/	MHA	Multiple *Pseudomonas aeruginosa* strains
Royintarat et al. (2019)	/	NA	*E. coli*
Schmidt et al. (2019)	/	TSA	*S. aureus* and *E. coli*
Shen et al. (2016)	/	LBA	*S. aureus*
Ukhtiyah et al. (2023)	/	MPN	*E. coli*
Xu et al. (2020)	PBS (after centrifugation)	YAPD	*Saccharomyces cerevisiae*

BHIA, Brain heart infusion agar; BPA, Baird-Parker agar; LBA, Luria−Bertani agar; MHA, Mueller Hinton Agar; NA, Nutrient Agar; PBS, Phosphate-Buffered Saline; PCA, Plate count agar; SSA, Salmonella Shigella agar; TSA, Tryptic Soy agar; YAPD, Yeast Extract Peptone Dextrose Agar.

Unlike fixed chemical agents, RONS are dynamic species whose reactivity depends on CAP device, volume of water and water environment (Lunder et al., 2025). Consequently, the suitability of traditional neutralizers and their capacity to effectively quench the oxidative potential of PAW, must be thoroughly evaluated. To date, no systematic studies have evaluated common neutralizers suitable for PAW testing. Despite the growing interest in CAP for water disinfection, there is currently no standardized protocol for neutralizing its residual antimicrobial effects. We have found that majority of PAW investigations have not incorporated neutralization procedures in either liquid or solid media. The absence of such steps allows the residual antibacterial activity of PAW to persist during microbiological assays, thereby continuing to exert effects on bacterial cells and potentially leading to an overestimation of its antimicrobial efficacy. Consequently, the establishment of standardized and validated protocols for the accurate and reproducible evaluation of PAW is urgently required. This lack of methodological consistency may lead to inaccurate estimations of CAP efficacy and hinder its comparison across studies. Establishing an effective neutralization protocol for PAW could support its future integration into more standardized antimicrobial testing frameworks, based on the approach used for chemical disinfectants. An appropriate neutralizer must meet three key criteria: it must effectively quench residual biocidal activity, exhibit no toxicity toward microorganisms, and not generate toxic by-products when combined with the antimicrobial agent (Sutton et al., 2002). Based on these requirements, commonly accepted neutralizers, PBS, NaCl with tryptone, polysorbate 80, lecithin, sodium thiosulphate were selected for evaluation. In addition, a combination of these neutralizers was assessed (Mix), as the use of mixed neutralizing agents is common in antimicrobial standards to achieve optimal neutralization performance. Their mechanisms of actions and main neutralization targets are presented in Table 2, but they have not been tested on PAW yet. Therefore, this study aims to (1) assess the prolonged antimicrobial activity of PAW using *S. aureus* and *E. coli* strains, (2) evaluate the neutralizing capacity of various chemical liquid and solid neutralizers on PAW, and (3) determine optimal culturing conditions for accurate PAW testing.

**TABLE 2:** Most common neutralizers and their mechanisms of action and neutralization targets.

Neutralizer	Mechanism of action	Main neutralization targets
PBS	Stabilization of pH (Scigiene, 2025)	Counteract acidification caused by RNS formation (Molina et al., 2013)
Sodium thiosulfate	Reduction of oxidants (Hayden and Goldsmith, 2010)	Unpaired electrons associated with RONS (Hayden and Goldsmith, 2010)
Lecithin	Ionic charges separation (Shah and Schulman, 1967) and emulsification (Youssef et al., 2024)	Free radicals (Pan et al., 2013)
Polysorbate 80	Emulsification and reduction of oxidative degradation (Mittag et al., 2022)	O_2_, HO^•^, O_2_^•–^, HOO^•^, H_2_O_2_, O (Mittag et al., 2022)
NaCl + tryptone	Reduces RONS-induced oxidative stress	H_2_O_2_, O_2_^•–^ (De Spiegeleer et al., 2004)

## 2 Materials and methods

### 2.1 PAW device

In this study, a DBD device was constructed using a flow-through circulating water system. A 500 mL volume of laboratory prepared hard water was continuously pumped through the system using a peristaltic pump (OEM201/YZ1515x pump head with DC24V-600RT motor unit, Chonry, China) at a flow rate of approx. 2 L/min. The water was directed as straight jet through the center of a cylindrical CAP reactor via a straight aluminum pipe (outer diameter 6 mm, inner diameter 4 mm, length ca. 10 cm), which reached approx. 15 mm into the reactor main body to serve as nozzle, while being connected to ground potential. The reactor’s main body was provided by a quartz tube (10 cm length, 20 mm outer diameter) wrapped with a copper mesh as high voltage (HV) electrode, positioned 25 mm below its top edge. The mesh was connected to a Fourier synthesis high voltage (HV) pulse generator (S/N 040-7, Ing.-Büro Jürgen Klein, Germany) delivering an HV pulses with alternating polarity, full width at half mean of 0.6 μs, pulse repetition rates of 0–30 kHz, pulse amplitudes of 5–20 kV, and corresponding pulse energies of 2.5–40 mJ. For stable operation with laboratory-prepared hard water, the HV pulse generator was operated with an amplitude of approx. 15 kV (amplitude setting 226 scale units) and a pulse repetition rate of 30 kHz (frequency setting 300 scale units), thereby generating a DBD plasma between the outer (mesh) HV electrode and the water jet serving as counter-electrode grounded via the aluminum nozzle. Safe operation in a laboratory setting was ensured by the reactor body being partially encased in epoxy resin (HERPELIN 1186, AMAL d.o.o., Ljubljana, Slovenia), such that the HV electrode mesh was covered by at least 10 mm epoxy in any given direction. The outlet of the reactor was connected to one neck of a three-neck round-bottom flask with bottom outlet, leaving the other two necks as access points for sampling and analyses, whereas the bottom outlet was directly connected to the peristaltic pump. Prior to each experiment, the reactor was rinsed with distilled water and disinfected with 5% hydrogen peroxide solution to minimize experimental errors and contamination. Furthermore, all experimental conditions including water and reactor temperatures were monitored to ensure best possible reproducibility of the treatment results.

### 2.2 Microorganism strains

Reference strains of microorganisms *Escherichia coli* NCTC 13351 and *Staphylococcus aureus* 43300, were obtained from National Collection of Type Cultures (United Kingdom) and Manassas (United States), respectively. The bacteria from the collection were transferred to Tryptic Soy Agar (TSA) (Biolife, Milan, Italy) and incubated at 37°C for 24 h.

### 2.3 CAP water treatment procedure

After overnight incubation the microorganisms were individually transferred from solid media into 0.9% NaCl solution, as described in Chapter 2.2. The bacterial concentrations were adjusted to 0.5 McFarland (1.5 × 10^8^ CFU/mL) for *E. coli* and 5 McFarland (15 × 10^8^ CFU/mL) for *S. aureus*. Each suspension was separately mixed into 500 mL of laboratory-prepared sterilized hard water, following the EN 1276 standard (EN 1276, 2020). The hard water consisted of 119 mg/L MgCl_2_ + 277.4 mg/L CaCl_2_ + 280.2 mg/L NaHCO_3_, and had a pH 8.3 and conductivity of 1,794 μS/cm. The final concentrations achieved with dilution were 1.5 × 10^6^ CFU/mL for *E. coli* and 15 × 10^6^ CFU/mL for *S. aureus*. The resulting contaminated water was then exposed to CAP treatment for 3 min, which had been previously determined as the sublethal exposure time for both strains. With a pulse energy input of 27.5 mJ and a repetition rate of 15 kHz, the power input delivered into the plasma reactor amounted to 825 W, thus providing a specific energy input for the total 3 min plasma treatment of 0.083 kWh/kg. Following treatment, 1 mL of PAW was collected from five points within the reactor and combined to form a representative composite sample. The experiments were conducted for both bacteria separately and in 3 replicates and 3 parallels, except for the chemical composition measurements which were conducted in 2 replicates and 4 parallels.

### 2.4 Effect of PAW exposure time on bacterial viability

To demonstrate the persistence of PAW’s bactericidal activity, bacterial viability was evaluated after 24 h of exposure without neutralization. Immediately after collection, 10 μL aliquots of PAW were inoculated onto TSA plates and incubated at 37°C for 24 h. To evaluate the bactericidal effect of long-lived RONS, 2.5 mL of PAW sample was transferred into a sterile container and stored at 4°C for 24 h. After overnight storage, the samples were again inoculated onto TSA plates in 10 μL aliquots and incubated under standard conditions. The control sample consisted of untreated water collected on the day of treatment. Validation of controls storage stability confirmed similar CFU values between day 0 and day 1 for both bacterial strains.

### 2.5 Time-dependent chemical composition of RONS in PAW

The concentrations of long-lived reactive oxygen and nitrogen species (RONS), specifically H_2_O_2_, O_3_, NO${}_{2}^{-}$, and NO${}_{3}^{-}$, were determined spectrophotometrically by Palintest Photometer 7500 (Palintest, United Kingdom) in accordance with the manufacturer’s instructions. These species were selected for analysis as they represent stable, long-lived products of CAP–liquid interactions and are commonly used as indicators of overall CAP-generated RONS chemistry, rather than as direct measures of short-lived reactive species. Hard water was prepared as described in Chapter 2.3., with one sample inoculated with both bacterial strains and one non-inoculated control. Both sample types were treated with CAP. Following the 3 min CAP treatment of water samples, 5 mL samples were collected from five different points within the reactor. Measurements were performed immediately after treatment in accordance with the manufacturer’s instructions. To evaluate the persistence of RONS, 12.5 mL of both treated and untreated samples was stored at 4°C for 24 h, after which the measurements were repeated using the same procedure.

### 2.6 Liquid neutralization

To evaluate the combined effect of dilution and chemical neutralization, 6 neutralizing agents were tested: (a) 1.15 g/L phosphate-buffered saline (PBS) solution, (b) 3 g/L sodium thiosulphate, (c) 3 g/L lecithin, (d) 30 g/L polysorbate 80, (e) 9 g/L sodium chloride + 1 g/L tryptone, and (f) a mixture of all the above components (Mix), each added at the same concentrations as when tested individually, ensuring that none of the neutralizers were diluted by the others (eq. 1 + 1 ratio). The concentrations of the neutralizing substances were selected according to the EN ISO 1276 standard (EN 1276, 2020). All neutralizers were prepared in sterilized distilled water.

Following the 3 min CAP treatment of water and sample collection, as described in section 2.3, 1 mL of the PAW was transferred into 9 mL of the respective neutralizer, as specified in antimicrobial testing standard EN 1276 (EN 1276, 2020). The suspensions were mixed immediately and kept at room temperature for 15 min contact time, with additional mixing performed midway and at the end of the contact time. Subsequently, 10 μL of each suspension was inoculated onto TSA plates and incubated at 37°C for 24 h. The analyses were conducted separately for each bacterial strain. Control samples were prepared without neutralization, using dilution in sterile hard water.

### 2.7 Solid media

Neutralization using solid media was also evaluated. Four commonly used solid media were selected for each bacterial strain, including both selective and non-selective types. This approach was chosen because some PAW-generating systems are not fully closed and may allow contamination, making selective media necessary. Neutralizing media were additionally tested to assess their ability to inactivate PAW. Both *E. coli* and *S. aureus* were tested on non-selective, non-neutralizing TSA and on non-selective, neutralizing Dey-Engley Agar (DEA). For *E. coli*, additional testing was performed on selective, non-neutralizing ENDO agar (EA) and Neutralizing ENDO agar (NEA), which was prepared by overlaying EA agar and Dey-Engley agar, with each medium used at its full prescribed concentration rather than diluted. Similarly, *S. aureus* was tested on selective Mannitol Salt Agar (MSA) and Neutralizing MSA (NMSA), prepared in the same additive manner (1 + 1 of MSA and DEA).

PAW containing bacteria was prepared as described in Chapter 2.3. A 10 μL aliquot of each sample was directly inoculated onto the respective solid medium and incubated at 37°C for 24 h. The control samples consisted of untreated water, pipetted directly onto the same type of agar medium.

### 2.8 Combined liquid/solid neutralization

To identify the most effective neutralization combination, the best-performing liquid neutralizer (MIX) from section 2.5 was tested in combination with all solid media described in section 2.6. PAW containing bacteria was prepared as outlined in section 2.3. Subsequently, 1 mL of the sample was mixed with 9 mL of the MIX neutralizer following the procedure detailed in section 2.5. A 10 μL aliquot of each resulting suspension was then inoculated onto the respective solid media and incubated at 37°C for 24 h. Results were expressed as log CFU/mL.

### 2.9 Statistical analysis

GraphPad Prism 8.4.3 software (GraphPad, United States) was used for statistical analysis. Normality was checked with the *t*-test (*p* > 0.05). To determine significant differences, the one-way analysis of variance was used *p* < 0.05 was considered significant.

## 3 Results

### 3.1 Storage effect on PAW antimicrobial potential

**FIGURE 1:**
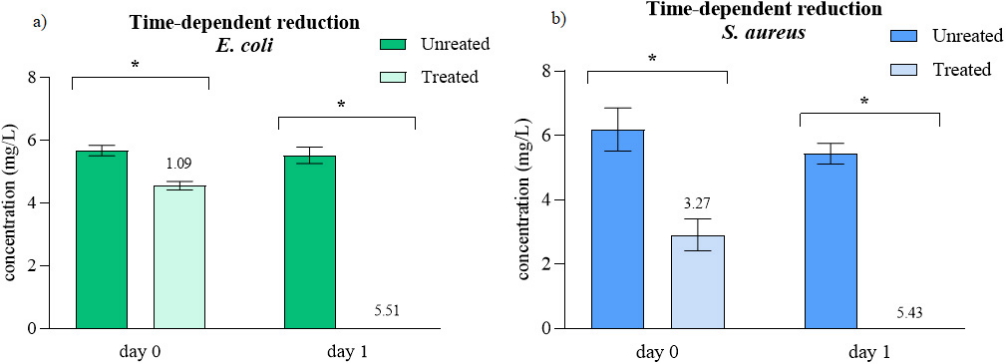
Storage effect of PAW on **(a)** *E. coli* **(b)** *S. aureus* viability in log CFU/mL. Reduction in log CFU/mL relative to control is written above columns. *Significant difference at *p* < 0.05.

With aim to assess PAW’s prolonged antimicrobial activity, tests were performed without neutralization immediately after treatment (day 0) and following 24 h of storage at 4°C (day 1). As shown in Figure 1a, the *E. coli* concentration decreased after 1 day of storage, resulting in a reduction of 4.42 CFU/mL compared to the values obtained on the day of treatment. A similar trend was observed for *S. aureus* (Figure 1b), where an additional 2.16 log CFU/mL decrease was recorded after 1 day of storage. In both figures, the log reduction values are indicated above each column, relative to the untreated control analyzed on each 0. The results are expressed as log CFU/mL.

### 3.2 Storage effects on PAW’s RONS

**FIGURE 2:**
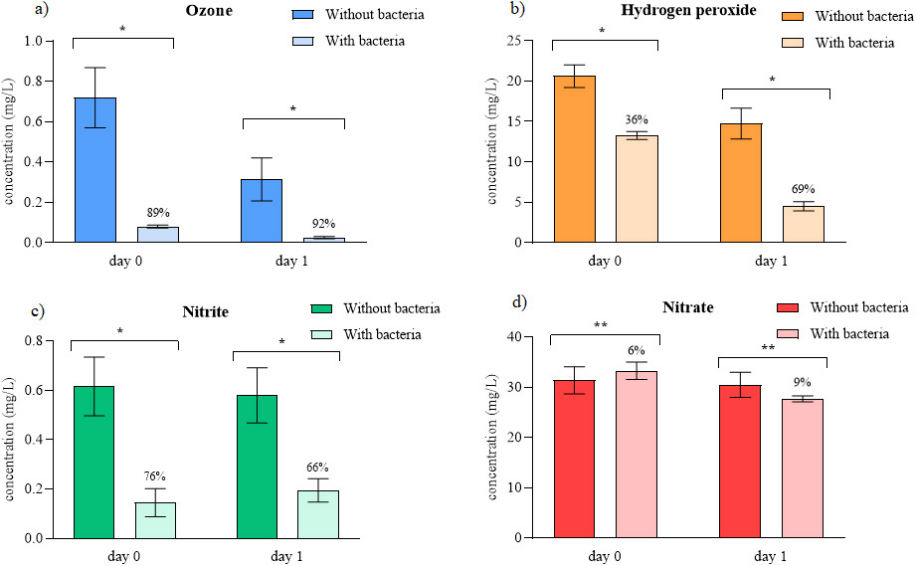
Storage effect on concentration of **(a)** ozone **(b)** hydrogen peroxide **(c)** nitrite **(d)** nitrate in PAW. Reduction in relative to control is written above columns. * Significant difference at *p* < 0.05, ** significant difference at *p* > 0.05.

To further analyze mechanisms of the prolonged antimicrobial activity of PAW, the concentrations of selected RONS, the main contributors to its sustained antimicrobial effect, were measured in samples with and without bacteria at day 0 and after 24 h of storage at 4°C. All selected RONS declined over time, though to different extents. Ozone (Figure 2a) decreased by 56% in PAW without bacteria and by 69% in samples with bacteria. Hydrogen peroxide (Figure 2b) showed similar behavior, with reductions of 28 and 66%, respectively. Nitrite (Figure 2c) showed only minor changes, decreasing slightly from 0.62 to 0.58 mg/L without bacteria and fluctuating from 0.15 to 0.20 mg/L with bacteria. Nitrate (Figure 2d) remained largely stable, decreasing from 31.40 to 30.49 mg/L in bacteria-free PAW and from 33.30 to 27.70 mg/L in samples with bacteria. Results are expressed in mg/L, with reductions indicated above each column.

### 3.3 Chemical neutralization

**FIGURE 3:**
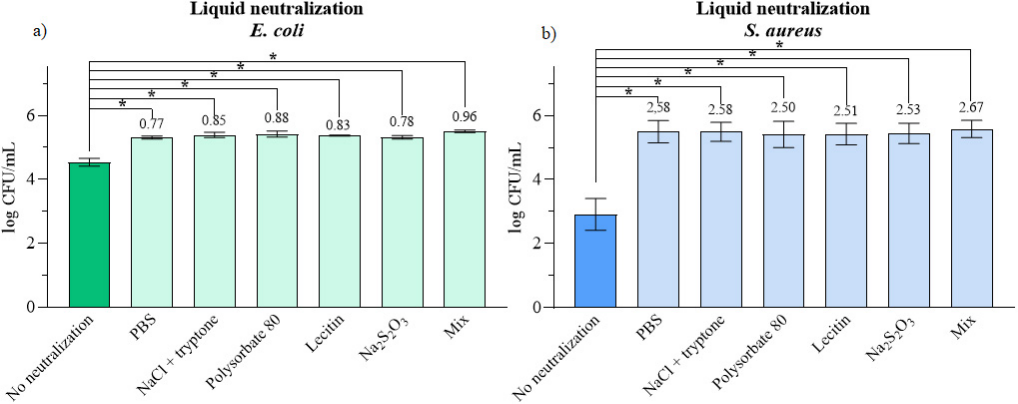
**(a)** Impact of chemical-dilution neutralization of PAW on *E. coli* **(b)** *S. aureus.* The increase relative to no neutralization control is written above columns. * Significant difference at *p* < 0.05.

The combined chemical and dilution neutralization method generally resulted in increased microbial growth, as the samples were transferred to the neutralizing solution immediately after treatment. Among the tested neutralizers, the Mix neutralizer demonstrated the highest efficiency in neutralizing PAW, showing a 2.67 log increase in *S. aureus* growth and a 0.96 log increase in *E. coli* compared to samples with no neutralization. However, no statistically significant differences (Supplementary Appendix 1) were observed among the different neutralizers for either bacterial strain. The comparison of neutralizers in experiments with *E. coli* and *S. aureus* is presented in Figures 3a,b, respectively. The log differences shown above each column represent the change in microbial growth relative to the non-neutralized control. The results are expressed as log CFU/mL.

### 3.4 Neutralization with incubating solid medium

**FIGURE 4:**
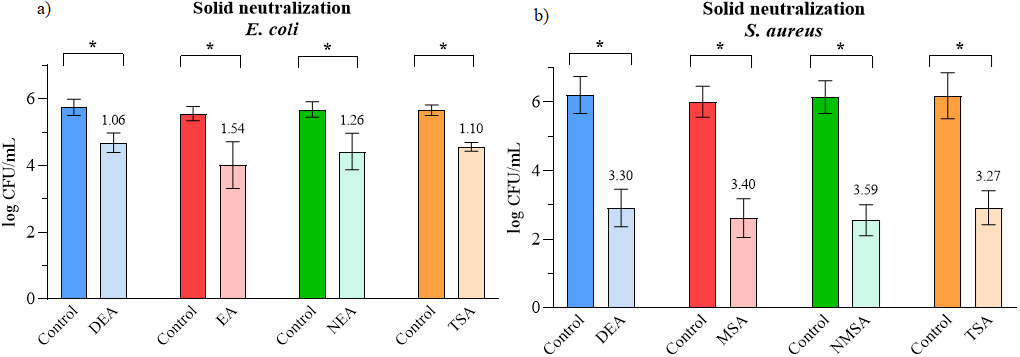
Impact of neutralization of PAW on solid medium on **(a)** *E. coli* **(b)** on *S. aureus.* Recovered number of cells expressed as log CFU/mL. Reduction in relative to control is written above columns. * Significant difference at *p* < 0.05.

To further assess the potential for PAW neutralization on solid media, four different agar types were tested for each bacterial strain. Results shown in Figures 4a,b are expressed as log CFU/mL for both untreated controls and treated samples on the same solid medium. A smaller difference in log values between the controls and treated samples, shown above each column, indicates a sufficient neutralization of PAW. For *E. coli*, the reduction in growth followed the order TSA/DEA < NEA < EA, while for *S. aureus* the trend was TSA < DEA < MSA < NMSA.

### 3.5 Combined neutralization

**FIGURE 5:**
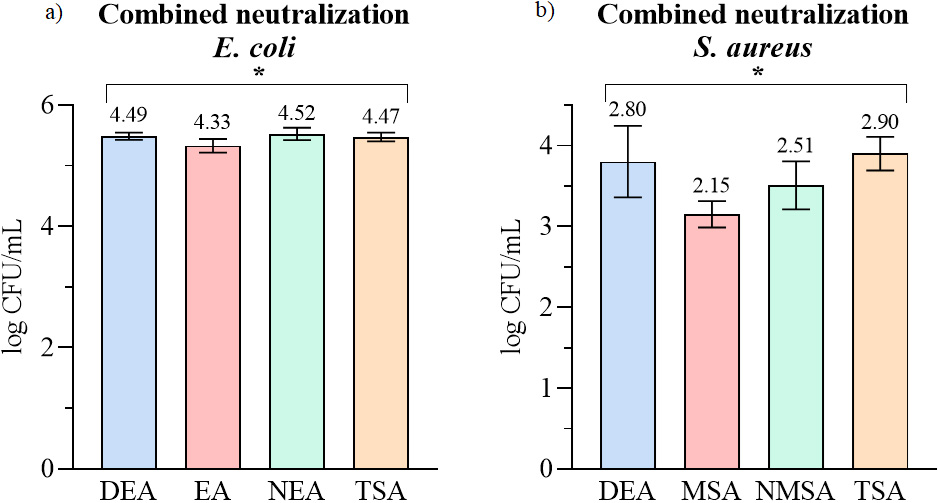
Impact of combined neutralization of PAW on **(a)** *E. coli* and **(b)** on *S. aureus.* Recovered number of cells expressed as log CFU/mL. Reduction in relative to control is written above columns. * Significant difference at *p* < 0.05.

To assess the best combination of liquid and solid neutralization, the best performing liquid neutralizer (Mix) was applied and tested on 4 different solid media. Results, presented in Figures 5a,b, are expressed as a log CFU/mL, with values indicated above each column. For *E. coli*, the effectiveness of the media followed the order TSA < DEA < NMSA < MSA, while for *S. aureus* the trend was NEA < DEA < TSA < EA, with no statistically significant differences (Supplementary Appendix 1) between mediums tested on the same bacterial strain.

## 4 Discussion

CAP has emerged as a promising novel water disinfection method (Oehmigen et al., 2010; Van Gils et al., 2013). The antimicrobial activity of CAP has been widely documented against various microorganisms, including *E. coli* (Machala et al., 2019; Nguyen et al., 2020; Hadinoto et al., 2021; Wang and Salvi, 2021), *S. aureus* (Schmidt et al., 2019; Shen et al., 2016; Han et al., 2016) and *Salmonella* spp. (Huang et al., 2018; Abdo et al., 2023; Boopathy et al., 2024). Our findings are consistent with these reports, confirming that CAP exhibits strong antimicrobial properties against both *E. coli* and *S. aureus* in water medium, achieving log reductions of 1.09 and 3.27, respectively, after 3 min of treatment. All comparisons were highly significant (*p* < 0.000001), confirming that the antimicrobial effect of PAW was not transient and was maintained within 24 h storage. Overall, treatment of *S. aureus* demonstrated higher log reductions than *E. coli*. Literature remains divided on this matter, as some studies report *S. aureus* as less susceptible to plasma activated PBS than *E. coli* (Zhao et al., 2020a; Togay et al., 2023). Conversely, other investigations reported no significant difference (Maisch et al., 2012) or even greater reductions of *S. aureus* (Klämpfl et al., 2012; Sedghizadeh et al., 2012) or Gram-positive bacteria in general (Rashmei et al., 2016). These inconsistencies likely reflect variations in plasma source, exposure time, and PAW composition. Although Rashmei et al. (2016) reported a much higher reduction of *E. coli* (8 log), their study used a 15 min CAP treatment of only 10 mL of water. Similarly, Kim et al. (2013) achieved a 5 log reduction of *E. coli* after 5 min when treating a water volume 10 times smaller than ours. Liew et al. (2023) also observed a high, 80% reduction of *S. aureus* after 3 min of CAP treatment of 5 mL of water. These comparisons confirm that the volume of treated water plays an important part in CAP disinfection efficiency and that studies are hard to be directly compared to each other. Notably, our study also used a flow-through system, and the others studied batch treatment. Another key determinant in microbial inactivation is also treatment duration that effects the bacterial reduction efficacy (Lunder et al., 2025). For example, F. Liu et al. (2010) achieved a total *S. aureus* reduction after 20 min of treatment, while Royintarat et al. (2019) achieved similar reductions for *S. aureus* in only 6.5 min of treatment. Furthermore, the design of the plasma device significantly affects the disinfection outcomes. In a study by Triantaphyllidou and Aggelopoulos (2025), a DBD system operating above water showed no significant reduction in *E. coli* even after 5 min of treatment. These differences were also confirmed by Tian et al. (2015), who showed better disinfection efficacy when CAP was produced directly under water. However, unlike most studies, our experimental setup was a flow-through system, with water serving as the second electrode in the DBD reactor. These observations highlight the importance of treatment parameters in antimicrobial testing. Beyond treatment conditions, understanding the post-treatment behavior of PAW is equally essential, as its antimicrobial activity can persist and evolve during storage.

Further experiments showed that storing the treated samples at 4°C for 24 h resulted in complete inactivation of both bacterial strains. This outcome reflects delayed loss of viability following PAW exposure rather than evidence of antimicrobial activity persisting throughout the entire storage period. Since bacteria were present during PAW production, the observed reduction to non-detectable levels after 24 h represents cumulative inactivation arising from immediate plasma effects and subsequent oxidative damage. Plasma-derived reactive species are known to induce oxidative stress in microbial cells, leading to damage of DNA, proteins, and lipids, which may result in immediate cell death or delayed loss of cultivability due to irreversible cellular injury (Xu et al., 2020). To specifically evaluate whether PAW retains antimicrobial activity after storage, an additional experiment was performed in which fresh bacterial suspensions were added to PAW after 24 h of storage (Supplementary Appendix 2). In this case, only modest reductions of 0.41 log CFU/mL for *E. coli* and 0.32 log CFU/mL for *S. aureus* were observed, indicating that antimicrobial activity decreases substantially over time. These findings demonstrate that while long-lived reactive species may persist in PAW after 24 h, their antimicrobial efficacy is markedly reduced compared to freshly generated PAW. Rashmei et al. (2016) similarly reported complete *E. coli* inactivation in CAP-treated PBS after 24 h of storage, observing an 8 log reduction between day 0 and day 1. In our study, a comparable effect was observed, with a total reduction in *E. coli* corresponding to a 5.51 log decrease. This finding reinforces the fact that PAW is a dynamic antimicrobial system rather than a static disinfectant, where chemical reactivity continues to contribute to microbial inactivation even after CAP treatment has ended. Han et al. (2016), also demonstrated a higher reduction after 24 h of storage following direct CAP treatment for both *E. coli* and *S. aureus*. Interestingly, they observed a noticeable decrease in bacterial CFUs even after only 1 h of CAP treatment of bacterial suspensions. This extended inactivation is primarily attributed to long-lived RONS generated in PAW. In terms of antimicrobial efficacy, PAW can be considered to exhibit residual activity. Chlorine behaves similarly: its strong residual effect requires neutralization, typically with sodium thiosulfate, which is routinely added to water-sample collection bottles (OECD and World Health Organization, 2003). This analogy further demonstrates that PAW must be neutralized upon sample collection to obtain accurate results. Without neutralization, RONS continue to inactivate bacteria during the incubation period, leading to overestimated antimicrobial efficacy.

To better understand their contribution to residual antimicrobial effect, we quantified H_2_O_2_, O_3_, NO_2_ and NO_3_ immediately after CAP treatment of water (day 0) and after 24 h storage (day 1) in samples with and without bacteria, respectively. These two experimental conditions are essential, as we directly evaluated how the presence of bacteria alters RONS composition immediately after treatment and after 1 day of storage. All of the measured RONS’ concentrations decreased within 24 h of storage, suggesting that they continued reacting with other reactive species or directly with bacteria (Dolezalova and Lukes, 2015). Importantly, PAW generated in the presence of bacteria consistently showed greater RONS depletion, indicating active bacterial consumption or degradation of both short- and long-lived species. Therefore, PAW effectiveness is decreased with the consumption of RONS, as they react with organic matter. This confirms that PAW composition is shaped not only by CAP chemistry but also by biological interactions occurring during and after treatment. Notably, even after 1 day of storage, RONS in PAW without bacteria remained at levels capable of depleting bacterial viability, further underscoring the need for effective neutralization.

Among all quantified RONS, ozone showed the most substantial decline during storage in both sterile and bacteria-containing PAW. Ozone decreased sharply after 1 day storage, with greater depletion in samples with bacteria (69%), confirming additional post-treatment reactions. When ozone reacts with bacteria (Van Gils et al., 2013), it degrades their cell walls and damages nucleic acids and carbon–nitrogen bonds of proteins (Baghal Asghari et al., 2021). Accordingly, ozone concentrations were significantly lower in samples containing bacteria on both days, reflecting its consumption during antimicrobial action. In sterile PAW, ozone decreased by 56% within 24 h. This decrease likely results from its reaction with water, producing additional hydroxyl radicals (Perinban et al., 2022) or from its interaction with HO*2* radicals to generate molecular oxygen (Rathore et al., 2021; Perinban et al., 2022). In studies by Perinban et al. (2022) and Han et al. (2016), the decrease was even greater, with ozone concentrations dropping below the detection limit after 24 h of storage time. The former study examined only PAW incubation, while the latter included analysis with bacterial presence. Similarly, Cai et al. (2023) reported an ozone reduction of over 50% after 24 h storage of PAW without bacteria, reaching levels comparable to those in our study, despite treating less than half the sample volume. Together, these comparisons highlight that ozone instability is consistently observed, but its magnitude varies with experimental parameters.

H_2_O_2_ is another key oxidant, which plays a crucial role in sustaining PAW’s antimicrobial action (Wong et al., 2023). It can decompose to produce hydroxyl radicals (Brisset and Pawlat, 2016), transform into hydroperoxyl radicals (Zhao et al., 2020b) or react directly with bacterial membranes, disrupting peptidoglycan layers, compromising the first layer of cell’s protection (Yusupov et al., 2013). In the present study, H_2_O_2_ concentrations were significantly lower (*p* < 0.000001) in samples containing bacteria, reflecting its consumption through these reactions. The H_2_O_2_ concentration also decreased by 29% after 1 day of storage in sterile PAW, whereas samples containing bacteria showed a far greater 66% reduction, indicating substantial consumption through bacterial reactions during post-treatment storage. A similar decrease of approximately 20% of H_2_O_2_ in PAW without bacteria was reported by Shen et al. (2016), although their experiments involved a smaller treated volume of water (15 mL). A decrease after 1 day of storage was also noted by Cai et al. (2023), reporting more than a one-fold reduction in H_2_O_2_ concentration when using a plasma jet device treating water without bacteria. In contrast, Vlad and Anghel (2017) did not observe a significant decline in H_2_O_2_ levels after 1 day of storage, which could be attributed to their longer treatment duration (50 min), potentially enhancing PAW stability. Importantly, none of these studies incubated PAW with bacteria, further emphasizing the novelty of our findings.

In contrast, nitrite and nitrate concentrations changed only minimally during storage, suggesting that their contribution to long term antibacterial activity was limited. However, nitrite levels were significantly lower in samples containing bacteria (*p* < 0.0001), whereas nitrate concentrations showed no significant difference between bacteria-containing (*p* = 0.23) and sterile (*p* = 0.06) groups. Nitrite’s stability in sterile PAW, paired with its reduction in bacteria-containing samples only on day 0, suggests that its primary reactivity occurs during the initial CAP exposure rather than during storage. While its levels could also be replenished through the reactions of NO with O*2* (Anderson et al., 2016) or OH radicals (Tachibana and Nakamura, 2019), such reactions appear to be confined to the immediate post-treatment period. Nitrate remained stable in both groups, with only a minor decrease in bacteria-containing PAW within 24 h. A decrease in nitrite concentration was also observed by Zhang et al. (2024) for 5 min CAP treatment of water without bacteria. Nitrate, however, only decreased when PAW was generated using a discharge power of 100 W or lower, emphasizing the influence of CAP generation parameters. Similarly, Shen et al. (2016) reported a reduction of approximately 7 mg/L in nitrite concentration after 1 day of storage, comparable to the 4 mg/L decrease observed in our study in PAW without bacteria. Consistent with our results, they found no significant changes in nitrate concentration. In a study by Vlad and Anghel (2017) the nitrate concentration remained stable after 1 day of storage in 35 mL water samples treated for 50 min with no bacteria present. Wang and Salvi (2021) also reported no decrease in nitrate over 24 h storage and a more than one-fold decrease in nitrite. However, their experiments were performed using PAW without bacteria and with distilled water at over twice smaller volumes, which may explain the observed differences in nitrite. Although RNS present in PAW are known to play a key role in bacterial inactivation (Graves, 2012; Zhou et al., 2020) our results suggest that ROS were primarily responsible for the observed antibacterial effects within the first 24 h. It is likely, however, that RNS act synergistically with ROS over longer storage times, contributing to bacterial nitrosative stress and maintaining PAW’s prolonged activity (Borkar et al., 2023).

Given this persistent RONS stability and consequent post-treatment reactivity, we hypothesized that neutralization should be performed immediately after exposure to CAP, following standard antimicrobial testing protocols (ASTM, 2002; EN 1276, 2020). Without neutralization, residual active compounds can continue to inhibit microbial recovery, leading to artificially high reductions and inaccurate results (Mehrgan et al., 2006). Despite its importance, the neutralization step has received little attention in published PAW research (Table 1), leading to inconsistent methodologies and potentially overestimated antimicrobial outcomes across studies. After reviewing the literature, we found that some studies (Machala et al., 2019; Schmidt et al., 2019; Maybin et al., 2023) skip the neutralization step altogether, while others (Wang and Salvi, 2021; Cai et al., 2023; Boopathy et al., 2024) only dilute the sample in different liquid mediums. The term “neutralization” is predominantly used in the field of plasma medicine, where it refers to the process of neutralizing CAP-generated ROS on various tissues. In this context, *N-*acetylcysteine is frequently employed as the neutralizing agent (Liu et al., 2024; Sun et al., 2024). However, this approach is not applicable to the present study, as *N*-acetylcysteine selectively neutralizes ROS but does not affect RNS (Ercan et al., 2016). Based on our review of their mechanisms of action (Table 2), the neutralizers most commonly specified in antimicrobial testing standards (EN 1276, 2020), PBS, NaCl with tryptone, polysorbate 80, lecithin, sodium thiosulphate, and a combined mixture (Mix), were selected for evaluation.

Chemical neutralization effectively neutralized PAW for both bacterial strains in all tested cases, as indicated by higher viability of bacteria in comparison to a sample where no neutralization was performed. The consistent high significance across all neutralizers (*p* < 0.0001) confirms that chemical components of PAW are essential for its antimicrobial effect. With the increased viability, the non-toxic nature of neutralizers and the quenching of bactericidal activity was also confirmed. Among the neutralizers, the “Mix” formulation yielded the best performance for both *E. coli* and *S. aureus*. This improvement likely arises from synergistic effects among its components. PBS and tryptone help maintain pH stability (Takahashl et al., 1997; Chen, 2011) while polysorbate 80, lecithin, and sodium thiosulphate act as surfactants and antioxidants that interact with diverse antimicrobial residues (Katerji et al., 2023). These compounds inhibit free radical formation and neutralize RONS (Das et al., 2022), collectively enhancing bacterial recovery. Similar conclusions were drawn by Katerji et al. (2023), who demonstrated effective neutralization of common antimicrobials such as parabens, ibuprofen, and sodium benzoate using neutralizers composed of polysorbate 80, lecithin, and sodium thiosulphate. Likewise, Espigares et al. (2003) achieved neutralization of peracetic acid with a formulation containing polysorbate 80, sodium thiosulphate, sodium bisulfate, and lecithin. However, the same formulation was ineffective against glutaraldehyde and o-phthalaldehyde, highlighting the importance of validating neutralization efficiency for each antimicrobial individually. In the context of PAW, this validation is particularly relevant due to its heterogeneous composition, which varies with plasma type, discharge power, and feed gas. While most of the cited studies examined combinations of neutralizing agents for chemical antimicrobials, our study investigated the effects of individual neutralizers, as such data have not been previously reported for PAW. Although differences among the neutralizers were not significant, the “Mix” formulation consistently supported higher microbial regrowth, reinforcing its suitability as the optimal choice for PAW neutralization. Notably, *S. aureus* exhibited a more pronounced difference between neutralized and non-neutralized samples compared to *E. coli*. This observation aligns with known differences in bacterial cell wall structures. Gram-negative bacteria like *E. coli* are more susceptible to extracellular oxidative damage due to their thinner peptidoglycan layer, while Gram-positive bacteria such as *S. aureus* possess a thicker peptidoglycan barrier that protects the cytoplasmic membrane (Lunder et al., 2025). Consequently, neutralizing agents are more effective in mitigating extracellular RONS damage, which is critical for *E. coli*, whereas in *S. aureus*, where intracellular oxidative damage predominates (Lim et al., 2025), the neutralizing effect appears to be less pronounced. For final validation of the “Mix” neutralizer, tests were performed in accordance with the EN 1276 standard (EN 1276, 2020). These validations confirmed the reliability of the laboratory procedure, the effectiveness of neutralization, and the non-toxic nature of the neutralizers, as detailed in Supplementary Appendix 3.

While chemical neutralization minimizes residual antimicrobial effects, the subsequent incubating environment on solid media can further influence bacterial enumeration. To further evaluate the neutralization capacity of solid media with different selectivity and neutralizing capacities were tested. Since some PAW-generation systems are not completely sterile, selective media were tested alongside non-selective and neutralizing media to ensure accurate recovery of bacteria. Previous research demonstrated that culture medium composition significantly influences bacterial recovery following stress (Noor et al., 2013). Four agar types were examined for each strain: TSA, DEA, EA, and NEA for *E. coli*; and TSA, DEA, MSA, and NMSA for *S. aureus*. TSA is a widely used non-selective agar. Its enzymatic digests of soy and casein support robust growth of both *E. coli* and *S. aureus* (HiMedia Laboratories, 2024b). To test the possibility of neutralization on solid media, non-selective DEA was also evaluated. Its ingredients include sodium thiosulfate and lecithin, which inactivate residual disinfectants and oxidative agents, aiding recovery of stressed or injured cells (Dey and Engley, 1983). Moreover, for selective growth in non-sterile environments, we tested EA and MSA, as they are known to inhibit the growth of unwanted organisms (Bonnet et al., 2020). MSA combines high salt concentration (7.5% NaCl) to inhibit most non-halotolerant bacteria with mannitol and phenol red, allowing selective growth of *S. aureus* and visual differentiation based on mannitol fermentation (Shields and Tsang, 2006). On the other hand, EA contains bile salts, basic fuchsin, and lactose, which inhibit Gram-positive organisms while differentiating lactose-fermenting coliforms like *E. coli* (HiMedia Laboratories, 2024a). However, it was shown that selective media can also suppress stressed or injured cells, leading to underestimation of viable counts (Bange et al., 2016). To address this, we also tested combinations of DEA with the respective selective agars (EA and MSA), following the approach of Bange et al. (2016), trying to achieve both neutralizing and selective capabilities of solid medium. In our experiments, selective agars generally yielded lower viable counts than non-selective ones, likely due to inhibition of PAW-injured cells, while neutralizing variants (NEA, NMSA) improved recovery but still lagged behind non-selective media (DEA, TSA). Similarly to our study, non-selective agar provided the most growth, followed by the selective agar in a study by Bange et al. (2016), who treated *S. aureus* with carvacrol. Furthermore, in a study by Grabow and Du Preez (1979) EA yielded the highest count of *E. coli* in comparison to some other coliform selective agars in river water samples. Additionally, Bange et al. (2016) found that injured *S. aureus* cells were effectively recovered by the selective agar (MSA) combination with non-selective media (TSA), and at a significantly higher concentration than recovery on selective media (MSA) alone. That’s contrary to our study, where NMSA had a higher difference to the controls than MSA. This could be explained by using different antimicrobial substances, as we used PAW and they used carvacrol.

Finally, to develop an optimized methodology for PAW testing, the most effective liquid neutralizer (Mix) was combined with each solid medium. The one-way ANOVA showed a statistically significant effect of agar type on PAW antimicrobial activity for both *E. coli* (*F* = 7.184, *p* = 0.001) and *S. aureus* (*F* = 8.291, *p* = 0.0007). In both bacterial strains, non-selective agars (TSA and DEA) again yielded the highest colony counts, while selective media (MSA and EA) produced lower recoveries. Selective formulations tend to restrict recovery of injured cells (Bange et al., 2016). Therefore, for routine PAW antimicrobial testing, we recommend combining chemical neutralization using the Mix formulation with non-selective or mildly neutralizing media such as NMSA for *S. aureus* and NEA for *E. coli* to achieve both effective neutralization and reliable microbial enumeration.

## 5 Conclusion

This study demonstrates that CAP represents an effective and promising approach for microbial inactivation in water disinfection applications. PAW generated in our flow-through DBD system exhibited strong antimicrobial activity against both *E. coli* and *S. aureus*, with the latter showing higher susceptibility under the tested conditions. The persistence of antimicrobial activity during storage, resulted in complete bacterial inactivation within 24 h at 4°C, highlighting the dynamic and evolving chemical reactivity of PAW, primarily governed by long-lived reactive oxygen and nitrogen species. Among these, hydrogen peroxide and ozone were identified as key contributors to post-treatment antimicrobial effects, whereas nitrite and nitrate exhibited relatively stable concentrations, suggesting a secondary or synergistic role in prolonged bacterial inhibition. Notably, PAW produced in the presence of bacteria exhibited consistently higher RONS losses, indicating that bacteria promote the consumption or breakdown of long-lived species.

Furthermore, the results emphasize the necessity of immediate neutralization following CAP exposure to ensure accurate antimicrobial evaluation. As recommended by different antimicrobial testing standards, the neutralizer should be applied immediately after antimicrobial (in our case PAW) treatment. Since different PAW devices produce varying chemical profiles, a single neutralizer may not suffice; instead, neutralization should involve multiple components tailored to quench the relevant reactive species. Importantly, the neutralizer should be validated to ensure it effectively quenches residual antimicrobial activity, is non-toxic to microorganisms, and does not generate harmful by-products when combined with the antimicrobial agent. The success of the validation process depends on the microorganisms used, PAW composition, and the liquid medium. In our case, the chemical-dilution method effectively quenched residual PAW activity, with the combined “Mix” neutralizer, consisting of PBS, NaCl with tryptone, polysorbate 80, lecithin, and sodium thiosulphate, proving most efficient. This mixture met the essential criteria for a suitable neutralizer. The validation process (described and presented in Supplementary Appendix 3) confirmed that the neutralizer was non-toxic to bacteria and possessed RONS quenching capacity. These properties are critical for ensuring accurate and reproducible antimicrobial testing. In evaluating incubating conditions, non-selective media, such as TSA and DEA, provided superior bacterial regrowth compared to selective agars, which tended to suppress injured cells. Therefore, for standardized PAW testing, we recommend integrating chemical neutralization with the Mix formulation and employing non-selective or mildly selective solid media (NEA for *E. coli* and NMSA for *S. aureus*).

These findings have important implications for the standardization of PAW antimicrobial testing, as the persistence of long-lived RONS highlights the need for immediate neutralization in experimental protocols to avoid overestimation of disinfection efficiency. In applied settings, the optimized neutralization protocol may improve the reliability of microbial quality assessments in CAP-treated water systems or industrial sterilization processes. Future studies using molecular or metabolic assays could further validate the neutralization process and its impact on bacterial viability.

## Data Availability

The datasets presented in this study can be found in online repositories. The names of the repository/repositories and accession number(s) can be found in the article/Supplementary material.
