# Supplementary material for: The power of neutralization: the critical step for the accurate antimicrobial potential of plasma-activated water
Source: Front Microbiol. 2026 Feb 20;17:1774713. doi: 10.3389/fmicb.2026.1774713 (PMC12962882; doi:10.3389/fmicb.2026.1774713)
Supplement: Supplementary file 1 [file Data_Sheet_1.pdf]

Supplementary Material

1 Appendix 1 – Statistical Analysis

Table 1: Statistical analysis of storage effect of PAW's antimicrobial potential on day 0 and day 1

Storage effect of PAW antimicrobial potential	Student's t test				
S. aureus	Mean of Unreated	Mean of Treated	Difference	t-ratio	p-value
day 0	6.188	2.913	3.274 ± 0.278	11.760	<0.000001
day 1	5.435	0.000	5.435 ± 0.161	33.690	<0.000001
E. coli					
day 0	5.666	4.546	1.120 ± 0.072	15.580	<0.000001
day 1	5.513	0.000	5.513 ± 0.133	41.490	<0.000001

Table 2: Statistical analysis of storage effect on RONS concentration on day 0 and day 1

Storage effect of PAW's RONS	Student's t test				
Ozone	Mean of Without bacteria	Mean of With bacteria	Difference	t-ratio	p-value
day 0	0.719	0.080	0.639 ± 0.076	8.370	0.000008
day 1	0.314	0.025	0.289 ± 0.055	5.295	0.00035
Hydrogen peroxide					
day 0	20.630	13.25	7.375 ± 0.741	9.959	0.000002
day 1	14.750	4.500	10.25 ± 0.997	10.280	0.000001
Nitrite					
day 0	0.616	0.145	0.471 ± 0.064	7.368	0.000024
day 1	0.580	0.195	0.385 ± 0.060	6.461	0.000072
Nitrate					
day 0	31.400	33.300	-1,900 ± 1.496	1.270	0.232965
day 1	30.490	27.700	2,786 ± 1.294	2.153	0.059734

Table 3: Statistical analysis of impact of chemical-dilution neutralization of PAW

Neutralization	Dunnett's multiple comparisons test	Mean No Neutralization	Mean Neutralization	Mean Difference	95,00% CI of difference	p-value
S. aureus	No neutralization vs. PBS	5.495	2.913	2.582	3.117 to 2.046	<0.0001
	No neutralization vs. NaCl + tryptone	5.495	2.913	2.582	3.117 to 2.046	<0.0001
	No neutralization vs. Polysorbate 80	5.412	2.913	2.498	3.034 to 1.963	<0.0001
	No neutralization vs. Lecitin	5.423	2.913	2.510	3.045 to 1.975	<0.0001
	No neutralization vs. $Na_2S_2O_3$	5.447	2.913	2.533	3.069 to 1.998	<0.0001
	No neutralization vs. Mix	5.583	2.913	2.670	3.205 to 2.135	<0.0001
E. coli	No neutralization vs. PBS	5.310	4.540	0.770	0.895 to 0.645	<0.0001
	No neutralization vs. NaCl + tryptone	5.392	4.540	0.850	0.969 to 0.734	<0.0001
	No neutralization vs. Polysorbate 80	5.418	4.540	0.878	0.996 to 0.761	<0.0001
	No neutralization vs. Lecitin	5.368	4.540	0.828	0.953 to 0.703	<0.0001
	No neutralization vs. $Na_2S_2O_3$	5.318	4.540	0.778	0.896 to 0.661	<0.0001
	No neutralization vs. Mix	5.506	4.540	0.966	1.09 to 0.841	<0.0001

Table 4: Statistical analysis of impact of neutralization of PAW on solid medium

Agars	Student's t test				
S. aureus	Mean Control	Mean Agar	Difference	t-value	p-value
DEA	6.207	2.908	3.299 ± 0.244	13.540	<0.0001
MSA	6.009	2.611	3.398 ± 0.233	14.580	<0.0001
NMSA	6.147	2.551	3.596 ± 0.214	16.840	<0.0001
TSA	6.188	2.913	3.274 ± 0.279	11.760	<0.0001
E. coli					
DEA	5.743	4.684	1.059 ± 0.115	9.190	<0.0001
EA	5.554	4.013	1.541 ± 0.232	6.650	<0.0001
NEA	5.679	4.422	1.257 ± 0.185	6.814	<0.0001
TSA	5.659	4.562	1.097 ± 0.060	18.360	<0.0001

Table 5: Statistical analysis of impact of neutralization of PAW on solid medium

Combination	ANOVA		
S. aureus	Mean	F value	p value
DEA	3.800	8.291	0.0007
MSA	3.150		
TSA	3.900		
NMSA	6.509		
E. coli			
DEA	5.488	7.184	0.001
EA	5.331		
TSA	5.475		
NEA	5.525		

2 Appendix 2 – PAW antimicrobial activity after 24 hours of storage

Methodology

PAW was prepared as described previously in Section 2.3, with the exception that no bacteria were present during plasma treatment. Following preparation, PAW was stored at 4 °C for 24 h. After storage, PAW was separately inoculated with *E. coli* and *S. aureus* to obtain final concentrations of 1.5 $\times 10^6$ CFU/mL and 15 $\times 10^6$ CFU/mL, respectively. The inoculated samples were maintained at room temperature for a contact time of 30 min. Subsequently, samples were serially diluted and plated as described in Section 2.4. Control samples consisted of untreated water inoculated with the same bacterial concentrations and processed under identical conditions.

Results and Discussion

To specifically assess whether PAW retains antimicrobial activity after storage, the experimental design excluded bacteria during plasma activation and introduced fresh bacterial suspensions after 24 h of storage. This approach was chosen to isolate the contribution of long-lived RONS present in PAW. The observed reductions of 0.41 log CFU/mL for *E. coli* and 0.32 log CFU/mL for *S. aureus* indicate that PAW retains measurable antimicrobial activity after 24 h of storage. Although the magnitude of reduction was modest compared to freshly prepared PAW, these findings suggest that long-lived RONS continue to contribute to bacterial inactivation even after extended storage. This observation is consistent with our findings in point 3.2. describing partial persistence of species such as hydrogen peroxide, nitrate, and nitrite, albeit at reduced concentrations over time.

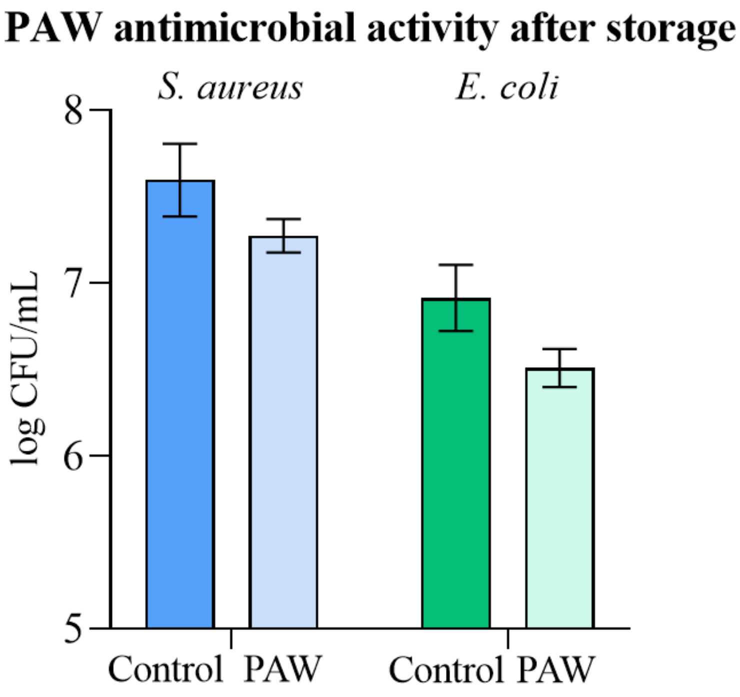

Figure 1: PAW antimicrobial activity after 24 h storage

3 Appendix 3 – Validation of Mix neutralizer

Methodology

To confirm the suitability of neutralizer, the best performing neutralizer (Mix) was chosen. It was tested for both tested bacterial strains respectively. Bacterial suspension was diluted in sodium chloride solution to assess baseline bacterial viability in the absence of hard water, neutralizer, or PAW.

Validation A (Hard water control): 9 mL of laboratory-prepared hard water (as described in Section 2.3) were mixed with 1 mL of the bacterial suspension, respectively. This condition was used to evaluate the effect of hard water on bacterial viability.

Validation B (Neutralizer toxicity assessment): 9 mL of tested neutralizer (Mix) were combined with 1 mL of the bacterial suspension. This validation step assessed whether the neutralizer exerted any toxic effects on the bacteria.

Validation C (Neutralization efficacy): 1 mL of PAW was mixed with 8 mL of neutralizer and was left to react for 5 min at room temperature. Following this neutralization period, 1 mL of bacterial suspension was added. This condition evaluated the ability of the neutralizer to effectively quench reactive species in PAW prior to bacterial exposure.

Following preparation, samples were diluted and plated onto TSA agar. After 24 h incubation at 37 °C, the colonies formed were counted and CFU/mL was calculated.

Results and Discussion

The results of the validation procedures are presented in Table 6. Across all validation conditions, the log CFU/mL values were comparable to those of the control, confirming the suitability of the neutralizer. Validation A demonstrated that the laboratory-prepared hard water provided appropriate experimental conditions, as bacterial counts were similar to the control. Validation B further confirmed that the neutralizer did not adversely affect bacterial viability, with log CFU/mL values comparable to the control. The RONS-quenching ability of the neutralizer was confirmed in Validation C, where bacteria were added after pre-neutralization of PAW with the Mix solution, resulting in bacterial counts comparable to the control.

Table 6: The validation of Mix neutralizer suitability

	VALIDATION (log CFU/mL)			Control
	A	B	C	
S. aureus	5.62	5.64	5.60	5.66
E. coli	5.06	4.99	5.05	5.17
